# Predictors of Serum 25-Hydroxyvitamin D Concentrations among a Sample of Egyptian Schoolchildren

**DOI:** 10.1155/2016/8175768

**Published:** 2016-01-28

**Authors:** Mones M. Abu Shady, Mai M. Youssef, Ebtissam M. Salah El-Din, Ola M. Abdel Samie, Hala S. Megahed, Samar M. E. Salem, Manal A. Mohsen, Ali Abdel Aziz, Safinaz El-Toukhy

**Affiliations:** ^1^Child Health Department, National Research Centre, Cairo, Egypt; ^2^Medical Biochemistry Department, Medical Division, National Research Centre, Cairo, Egypt

## Abstract

*Objective*. To assess the level of 25-hydroxyvitamin D status among a sample of Egyptian schoolchildren and to evaluate predictors of deficiency and insufficiency.* Subjects and Methods*. A cross-sectional study comprising 200 prepubescent schoolchildren aged from 9 to 11 years was performed. A questionnaire including frequency of midday sun exposure, milk intake, physical activity, and level of maternal education was taken. Body mass index (BMI) was calculated; serum 25-hydroxyvitamin D [25(OH)D], serum calcium, phosphorus, and parathyroid hormone were measured.* Results*. Vitamin D deficiency [serum 25(OH)D < 20 ng/mL] was detected in 11.5% of subjects while its insufficiency (serum 25(OH)D is between 20 and 29.9 ng/mL) was detected in 15%. Results revealed that obesity, low physical activity, low sun exposure, and low maternal education level are significant predictors of insufficiency, though female gender, low maternal education level, and low milk intake are significant predictors of deficiency. Lower serum phosphorus and higher serum parathyroid hormone were significantly associated with both deficiency and insufficiency (*p* < 0.05).* Conclusion*. Vitamin D deficiency and insufficiency are common among schoolchildren in Egypt. Food fortification, vitamin D supplementation, and increasing maternal awareness about the importance of physical activity and exposure of their children to ultraviolet light may help to overcome this problem.

## 1. Introduction 

Vitamin D status influences the absorption of calcium and phosphate from the gut and is known mainly for its role in bone health throughout life. During periods of rapid growth in childhood, vitamin D deficiency causes growth retardation, skeletal deformities, and an increased fracture risk later on in life [1]. Emerging evidence suggests that vitamin D plays an important role in the modulation of the immune responses. In addition, vitamin D has been recognized as an anti-infective agent due to its effects on the innate and adaptive immune responses [2]. Low blood levels of vitamin D have been associated with an increased risk of hypertension [3], myocardial infarction [4], sudden cardiac death [5], diabetes [6], obesity [7], and cancer [8, 9]. Vitamin D deficiency and insufficiency are still reported as frequent problems in children and adolescents worldwide. Surprisingly it is most notable in the Middle East, in spite of its prevailing sunny weather [10, 11]. Factors known to influence vitamin D status include sunshine exposure, skin pigmentation, seasonality, body mass index (BMI), physical activity, and dietary factors, particularly vitamin D-rich food or fortified milk [12–14]. The serum 25-hydroxyvitamin D (25(OH)D) concentration has been accepted as the primary determinant of vitamin D status, and many studies reported concentrations of this metabolite in children [15]. As few foods contain vitamin D, guidelines recommended supplementation through a suggested daily intake and tolerable upper limit levels. It has been also suggested to measure the serum 25-hydroxyvitamin D level as the initial diagnostic test in patients at risk of deficiency. Treatment with either vitamin D2 or vitamin D3 is recommended for deficient patients [16].

To the best of our knowledge, a limited number of reports on vitamin D status and its risk factors in the Egyptian pediatric population have been published to date. Accordingly, the objectives of the present study were to assess vitamin D status on basis of serum 25(OH)D concentrations and its relation to PTH in a sample of Egyptian schoolchildren and to identify the predictors for vitamin D deficiency and insufficiency.

## 2. Subjects and Methods

This cross-sectional study comprised 200 children (98 boys and 102 girls). They were prepubescent (Tanner stage 1) aged from 9 to 11 years. The pupils were recruited randomly from two primary public schools situated in Giza governorate in Egypt. Although there is abundant sunlight in Egypt almost throughout the year, all subjects were studied during the same period (April and May) to avoid the effect of seasonal variation on serum 25-hydroxyvitamin D levels. Children excluded were those below 9 or over 11 years, known to have an endocrinal or genetic obesity, with chronic debilitating diseases (e.g., hepatic or renal disease, diabetes mellitus, rheumatic and congenital heart diseases, hypertension, and chronic lung diseases), with metabolic diseases (metabolic rickets and calcium metabolic disorders), with malabsorptive disorders (Crohn's disease, cystic fibrosis, and celiac disease), having cancer, who have taken medications as anticonvulsants or systemic glucocorticoids, and, finally, all who are taking calcium, vitamin D, or multivitamin supplements.

The children were asked about frequency of fortified milk intake/day and this was categorized as once or more per day and less than once per day. The questionnaire also sought information about hours of midday sun exposure/week and physical activity. Physical activity information was reported and it included frequency, type of activities performed, and duration (number of hours per week) and was categorized as high (≥4 hours/week) and low (<4 hours/week). Maternal educational level was assessed by direct questions to the parents and was categorized as high level (postsecondary school) and low level (secondary school or less) [17].

### 2.1. Physical Examination

Children were subjected to thorough clinical examination that included chest, heart, abdominal, and central nervous system examination. Assessment of pubertal development was done according to Tanner's scoring [18].

Anthropometric measurements, including weight and height, were performed. The height was measured to the nearest 0.1 cm using a Holtain portable anthropometer, and the weight was determined to the nearest 0.01 kg using a Seca Scale Balance with the subject dressed in minimal clothes and without shoes. The BMI was calculated as weight (in kilograms) divided by height (in meters) squared. The BMI *Z*-score was calculated based on the WHO growth standards with the help of AnthroPlus Program of PC [19].

### 2.2. Laboratory Determinations

A venous blood sample was withdrawn from each child after an overnight fast of 12 hr. Serum was separated and stored at −20°C until assayed. Serum samples were assayed for 25-hydroxyvitamin D [25(OH)D] using quantitative enzyme immunoassay [20]. Serum 25-hydroxyvitamin D [25(OH)D] is the major circulating form of vitamin D and a standard indicator of vitamin D status [21]. The 25(OH)D cutoffs to define deficiency and insufficiency vary and have most recently been framed by the 2011 desirable levels of the Institute of Medicine Report and the Endocrine Society Guidelines [22, 23]. According to these guidelines, vitamin D deficiency was defined in the present study as a concentration of 25(OH)D < 20 ng/mL and that between 20 and 29.9 ng/mL as insufficiency and a concentration ≥ 30 ng/mL as sufficient. Human Parathormone (hPTH) was measured using quantitative enzyme immunoassay according to Bouillon et al. [24]. In addition, calcium and phosphorus were assayed in serum according to Goodwin et al. [25, 26].

### 2.3. Statistical Analysis

All statistical analyses were carried out using the SPSS (Statistical Package of Social Sciences, Chicago, IL, USA) for Windows software program version 16.1. Continuous data were expressed as mean ± SD and were compared using Student's *t*-test. Categorical data were expressed as frequencies and percentages. Pearson's correlation analysis was carried out to evaluate the association between continuous exposure and continuous covariates. A multiple logistic regression analysis was performed to study the association between presumed risk factors and vitamin D status. *p* value less than 0.05 was considered as statistically significant.

## 3. Results

The mean age of all subjects studied was 10.39  ±  0.58 years (age ranged from 9 to 11 years). They were 98 males (49%) and 102 females (51%). The number of obese subjects (children with BMI-for-age *Z*-score is > 2 whether boys or girls) was 88 (44%) and that of nonobese subjects was 112 (56%). The mean serum level of 25(OH)D of the enrolled children was 41.25 ± 13.95 ng/mL (range: 15–65 ng/mL). Vitamin D deficiency was detected in 23 subjects (11.5%) while vitamin D insufficiency was found in 30 subjects (15%). So serum 25(OH)D < 30 ng/mL was found in 53 subjects (26.5%).

Table 1 shows serum 25(OH)D in relation to studied variables presumed precarious for vitamin D insufficiency. Females, obese children, children of low educated level mothers, children with low physical activity, and children with milk intake less than once/day had statistically significant lower serum 25(OH)D than their peers (*p* < 0.005).  Table 2 shows that the concentration of 25(OH)D was significantly negatively correlated with BMI, serum calcium, phosphorus, and parathyroid hormone (*p* < 0.05). No significant correlation was detected between 25(OH)D and the age of the studied children.

Tables 3 and 4 show the logistic regression analysis for the presumed predictors of vitamin D status. Females were 10.3 times more likely to have vitamin D deficiency than males (*p* < 0.05). However, no significant effect of gender was detected on vitamin D insufficiency (*p* > 0.05). Obese children were 5.1 times more likely to have vitamin D insufficiency only than nonobese (*p* < 0.05), while no significant effect of obesity was detected on vitamin D deficiency (*p* > 0.05). Children of low educated mothers had significantly increased risk of having both vitamin D deficiency and insufficiency compared to children of mothers with high education (*p* < 0.05). Children with low physical activity were 3.6 times more likely to have only vitamin D insufficiency than those with high physical activity (*p* < 0.05). Children with milk intake less than once per day had significantly increased risk of having only vitamin D deficiency compared to those with milk intake once or more per day (*p* < 0.05). Moreover, only vitamin D insufficiency was significantly associated with low hours of sun exposure per week (*p* < 0.05). As regards serum calcium, no significant association was detected between it and either vitamin D deficiency or insufficiency (*p* > 0.05). Lower serum phosphorus and higher serum parathyroid hormone were significantly associated with both vitamin D deficiency and insufficiency (*p* < 0.05).

## 4. Discussion

Vitamin D deficiency or insufficiency is a very common health problem in children and adolescents. Emerging evidence suggests a close association between 25(OH)D levels and immune function, insulin resistance, and cardiometabolic diseases, all of which drive the consequences of vitamin D deficiency beyond those involving mineral and bone metabolism.

A relatively high prevalence (11.5%) of vitamin D deficiency (serum 25(OH)D < 20 ng/mL) was identified in the present study among the apparently healthy, minor group of school-aged children. An even larger proportion (26.5%) had serum 25(OH)D levels below the threshold (<30 ng/mL) which is becoming increasingly accepted as optimal for bone health and protection against malignancies, infections, and other diseases. The risk factors for vitamin D insufficiency or deficiency in the present study were female gender, low physical activity, obesity, low milk consumption, low level of maternal education, and few hours of sun exposure. Serum 25(OH)D levels were inversely related to PTH and serum phosphorus. Although serum calcium was inversely related to 25(OH)D levels, low serum calcium was not identified to be a risk factor to vitamin D deficiency or insufficiency in logistic regression.

Our results show a serum 25(OH)D < 30 ng/mL found in 53 subjects (26.5%) and a prevalence of vitamin D insufficiency among healthy children of 15%, which are lower than other reports from other countries around the world (49.4% in Italy [27], 39% in Europe [11], 56.4% in USA [28], and 51.4% in Korea [29]). The difference may be due to the sunny climate of Egypt compared with that in Europe and USA.

In the present study, boys had a statistically significantly higher serum 25(OH)D than girls who had a significantly increased risk of vitamin D deficiency; this is in agreement with Houghton et al. [30] and Habibesadat et al. [31]. The difference in gender may be due to lifestyle factors such as spending more time indoors, less time outdoors, and coverage of skin by clothing that could affect cutaneous synthesis of vitamin D.

In the present study a significant negative correlation was reported between BMI and serum 25(OH)D levels, where the increased BMI was a risk factor for vitamin D insufficiency. Overweight and obese subjects have a high prevalence of vitamin D deficiency. These findings may be explained by sequestration of the fat-soluble vitamin D within adipose tissues. Moreover, adiponectin has been recently identified as a key plasma protein that links vitamin D deficiency to pediatric obesity [32]. These findings are in agreement with Wortsman et al. [33], Youssef et al. [34], Valtueña et al. [13], and Kao et al. [35] and contradictory to Cole et al. [36].

Lack of exposure to sunlight and low physical activity were relevant risk factors for vitamin D insufficiency in the present study, which is in accordance with Bener et al. [37] who studied the factors associated with vitamin D deficiency in Qatari population. The same results were found in one study done on Chinese adolescents [38] and another one on US adolescents [28]. 25(OH)D deficiency was highly prevalent among children in Southern Iran [39]. It was related to insufficient sun exposure, low physical activity, advancing age, and pubertal stage.

An association between children's 25(OH)D deficiency and low mother's educational levels was detected in the present study. This result may be attributed to a lower diet quality accompanied with low educational level which is in agreement with the study of Voortman et al. [40] in Netherlands who also found that vitamin D deficiency was associated with low household income (OR: 1.74; 95% CI: 1.34, 2.27 for low versus high income) and low diet quality. In addition, Li et al. [41] found that vitamin D deficiency was much more associated with female gender, high body mass index, and lower socioprofessional status. In contrast to our result, Wakayo et al. [42] found that the educational status of mothers of children enrolled in their study had a statistically significant association with their children vitamin D status, where children whose mothers had formal education were found to be more likely to develop vitamin D deficiency compared to those whose mothers did not have a formal education (after adjusting for study setting). They attributed their results to the tendency to avoid direct sun exposure by the more educated mothers through staying indoors or using sun screens, for themselves and their children as much as possible to avoid skin cancer risk or merely to avoid sun burns or excessive tanning. In contrast, researchers from the USA [43] and Middle East [37] reported no association between maternal education and their children vitamin D status.

A prospective study of 98 rachitic and 50 controls (aged 6 months to 4 yr) from university and community outpatient hospitals in Egypt and Turkey was done by Baroncelli et al. [44]. Positive correlations were found between serum calcium and serum 25(OH)D (*r* = 0.23, *p* < 0.05) which was in contrast to our study which showed a negative correlation between serum 25(OH)D and serum calcium (*r* = −0.278, *p* < 0.001). This could be explained on the basis of secondary hyperparathyroidism in response to vitamin D deficiency. In cases of prolonged vitamin D deficiency the parathyroid glands increase the PTH production in order to increase calcium levels by “stealing” it from the bones. Therefore, people with vitamin D deficiency might have normal blood calcium levels and a high PTH level as a result of secondary hyperparathyroidism [45].

In agreement with the present study, serum 25(OH)D levels correlated negatively with serum PTH levels in a study done by Baroncelli et al. [44] (*r* = −0.29, *p* < 0.01). In addition, Carpenter et al. [46] and Arabi et al. [47] detected a significant negative association between 25(OH)D and parathyroid hormone in multivariate analysis, which is in accordance with the results of the present study.

This study has several notable limitations including the cross-sectional design, a narrow age range, and small sample size. The findings, however, emphasize the need for nationally representative data to appropriately assess the magnitude of vitamin D deficiency in different areas in Egypt.

*Conclusion*. The present study demonstrated that Egyptian children are at a significant risk of vitamin D deficiency and insufficiency. Female gender, high BMI, low mother educational level, low physical activity, low milk intake, and low sun exposure are all significant risk factors of vitamin D deficiency or insufficiency. Moreover, children with vitamin D deficiency and insufficiency may develop secondary hyperparathyroidism that may affect bone mass accrual. Future studies should employ measures to appropriately quantify sun exposure and outdoor activity and to assess the deleterious effect of vitamin D deficiency on bone mineral homeostasis of growing children in Egypt during their most critical period of bone development. From a perspective of integrative childhood care, it is recommended to spend more time in outdoor activity for sunlight exposure and increased vitamin D supplementation alongside interventions to increase physical activity and reduce sedentary behavior to achieve healthier lifestyle in higher risk children.

## Figures and Tables

**Table 1:** Serum 25(OH)D levels related to presumed risk factors for vitamin D insufficiency.

Variable	25(OH)D	*t*-test	*p*
	Mean ± SD		
Gender			
Male	43.570 ± 14.192	2.426	0.016^*∗*^
Female	38.842 ± 13.336		
BMI			
Normal	48.205 ± 10.511	9.604	<0.001^*∗*^
Obese	32.406 ± 12.752		
Mother education			
High	44.428 ± 10.360	2.554	0.011^*∗*^
Low	39.308 ± 15.468		
Physical activity			
High (≥4 hours/week)	47.138 ± 12.282	4.538	<0.001^*∗*^
Low (<4 hours/week)	38.154 ± 13.814		
Fortified milk intake			
Once or more/day	44.227 ± 12.542	4.184	0.002^*∗*^
Less than once/day	35.967 ± 14.819		

^*∗*^ *p* < 0.05 is significant. BMI: body mass index.

**Table 2:** Correlation between serum vitamin D (25(OH)D) concentration and different predictive variables.

Variable		Concentration of 25 (OH)D (ng/mL)
Age (years)	*r*	0.074
	*p*	0.299
BMI	*r*	−0.636
	*p*	<0.001^*∗*^
Hours of sun exposure/week	*r*	0.665
	*p*	<0.001^*∗*^
Serum calcium (mg/dL)	*r*	−0.278
	*p*	<0.001^*∗*^
Serum phosphorus (mg/dL)	*r*	−0.156
	*p*	0.028^*∗*^
PTH (pg/mL)	*r*	−0.577
	*p*	<0.001^*∗*^

^*∗*^ *p* < 0.05 is significant.

BMI: body mass index.

PTH: parathyroid hormone.

**Table 3:** Logistic regression for presumed risk factors for vitamin D deficiency (25(OH)D < 20 ng/mL).

	*B*	SE	OR	95% CI for OR		*p*
				Lower	Upper	
Gender Male versus female	2.331	1.024	10.291	1.382	76.610	0.023^*∗*^
BMI Normal versus obese	0.783	1.073	2.188	0.267	17.934	0.466
Mother education Postsecondary versus secondary school or less	3.096	1.137	22.112	2.380	205.476	0.006^*∗*^
Physical activity High (≥4 hours/week) versus low (<4 hours/week)	1.516	0.805	4.552	0.940	22.043	0.060
Fortified milk intake Once or more/day versus less than once/day	1.212	0.562	3.360	1.116	10.114	0.031^*∗*^
Sun exposure Hours/week	−0.131	0.216	0.877	0.574	1.339	0.543
Serum calcium	0.512	0.482	1.668	0.649	4.291	0.288
Serum phosphorus	−0.460	0.213	0.631	0.416	0.959	0.031^*∗*^
PTH	0.058	0.018	1.060	1.023	1.099	0.001^*∗*^

^*∗*^ *p* < 0.05 is significant.

BMI: body mass index, OR: odds ratio.

PTH: parathyroid hormone, CI: confidence interval.

**Table 4:** Logistic regression for presumed risk factors for vitamin D insufficiency (25(OH)D 20–29.9 ng/mL).

	*B*	SE	OR	95% CI for OR		*p*
				Lower	Upper	
Gender Male versus female	1.050	0.574	2.859	0.927	8.814	0.068
BMI Normal versus obese	1.630	0.790	5.103	1.084	24.013	0.039^*∗*^
Mother education Postsecondary versus secondary school or less	2.785	0.705	16.194	4.064	64.530	<0.001^*∗*^
Physical activity High (≥4 hours/week) versus low (<4 hours/week)	1.270	0.556	3.561	1.198	10.582	0.022^*∗*^
Fortified milk intake Once or more/day versus less than once/day	0.185	0.447	1.203	0.501	2.890	0.679
Sun exposure Hours/week	−0.538	0.207	0.584	0.389	0.877	0.009^*∗*^
Serum calcium	0.064	0.406	1.066	0.481	2.364	0.875
Serum phosphorus	−0.500	0.160	0.606	0.443	0.829	0.002^*∗*^
PTH	0.050	0.014	1.051	1.024	1.080	<0.001^*∗*^

^*∗*^ *p* < 0.05 is significant.

BMI: body mass index, OR: odds ratio.

PTH: parathyroid hormone, CI: confidence interval.
